# *Rhododendron minus* seedlings achieve similar performance across light environments with anthocyanin accumulation and architectural adjustments under light stress

**DOI:** 10.1093/aobpla/plag008

**Published:** 2026-02-13

**Authors:** Miranda K Shetzer, Emma Farley, Juliana S Medeiros

**Affiliations:** Research Department, Holden Arboretum, 9500 Sperry Rd, Kirtland, OH 44094, United States; Department of Biology, Case Western Reserve University, 10900 Euclid Ave, Cleveland, OH 44106, United States; Research Department, Holden Arboretum, 9500 Sperry Rd, Kirtland, OH 44094, United States; Research Department, Holden Arboretum, 9500 Sperry Rd, Kirtland, OH 44094, United States

**Keywords:** *Rhododendron minus*, light intensity, photoprotection, anthocyanins, plant architecture, intraspecific variation, provenance, climate

## Abstract

Influences of climate change on light availability are often overlooked; yet, understory species may experience shifts in irradiance as rising temperatures influence phenology and community composition. Light management is crucial for seedling success, and a whole-plant approach can help elucidate consequences of light on plant performance. *Rhododendron minus* is an evergreen shrub native to the Southeast United States that grows from rock outcrops to the understory. We conducted two experiments to unravel influences of light on plant function: (i) a manipulative greenhouse experiment on seedlings from a sun-exposed provenance examining pigments, plant architecture, and biomass patterns under shade, ambient, and supplemental light and (ii) a common garden experiment comparing pigments of mature plants from six provenances differing in latitude, elevation, climate, and solar radiation. We used multispectral imaging to estimate anthocyanin through the normalized difference anthocyanin index (NDAI) and chlorophyll through the normalized difference vegetation index (NDVI). Supplemental light seedlings had significantly higher NDAI than shade and ambient seedlings, but there was no significant treatment effect on NDVI or total biomass. Supplemental light seedlings also exhibited leaf movements that reduced projected surface area over time. This work highlights the importance of anthocyanins and plant architecture in allowing seedlings to maintain similar performance across light environments. In our common garden experiment, plants from northern, colder provenances had higher NDAI compared to warmer, southern provenances. We suggest that interactions between temperature and irradiance likely drive intraspecific variation in NDAI across the range, indicating that climate change could influence future pigment evolution.

## Teaser

Rhododendron minus plants growing in exposed, cold habitats of the Appalachian Mountains may benefit from higher anthocyanin due to combined pressures of high light and low temperature during winter months. In addition to anthocyanins, architectural adjustments may help plants balance light interception with metabolic capacity. Plants from the southern region of the species range had less anthocyanin and may instead use carotenoids as a photoprotection mechanism.

## Introduction

Climate change is expected to cause local extinctions and recolonizations of plant species that can affect community composition of the forest overstory (García-Valdés *et al.* 2018, Brice *et al.* 2019, Schippers *et al.* 2021). As biodiversity patterns continue to shift, understory shrubs could experience changes in light availability, leading to seed germination in suboptimal light environments and possible seedling mortality (Lin *et al.* 2014). Furthermore, changes in temperature are leading to shifts in phenology (Geng *et al.* 2020, Inouye 2022), affecting the timing of leaf bursts and ultimately light intensity beneath tree canopies (Xiong *et al.* 2024). It is well known that light availability influences functions imperative for growth and survival (Beckage and Clark 2003), notably photosynthesis (Mathur *et al.* 2018, Zhang *et al.* 2022); yet, the synergy between climate change and light availability on whole-plant function remains largely unknown. This hinders the ability to predict ecological outcomes of changing environments. The shrub growth form is thought to be advantageous for survival in stressful environments, but shrubs have historically been neglected in plant research (Götmark *et al.* 2016). Understory shrubs have great importance to ecosystem processes, including nutrient cycling and tree seedling establishment (Qiao *et al.* 2014, Gavinet *et al.* 2016), making it imperative to investigate how stress responses determine shrub performance.

Light is a highly heterogeneous resource, with temporal variation resulting from diurnal and seasonal changes in light intensity and spatial variation resulting from differences in the degree of shading from the overstory and neighbouring plants (Pearcy 2007). Photosynthetic pigments are essential for light management and are highly responsive to changes in irradiance (Krause *et al.* 2012, Ouzounis *et al.* 2015, Hazrati *et al.* 2016, Mathur *et al.* 2018). Chlorophyll is the main pigment responsible for light harvesting during photosynthesis, and increased chlorophyll under shade is often associated with enhanced photon absorption (Naves *et al.* 2018, Stewart *et al.* 2020). Light is essential for carbohydrate synthesis, but if light absorption exceeds metabolic capacity, then the photosynthetic apparatus can become overexcited, leading to increased production of reactive oxygen species (ROS) and subsequent oxidative damage that could compromise photosynthetic integrity (Pospíšil 2016, Gu *et al.* 2017, Bassi and Dall’Osto 2021).

Anthocyanins are a family of pigments contributing to red hues in plants that have been suggested to play a role in ROS scavenging under multiple abiotic stressors, including high light, drought, and low temperature (Xu *et al.* 2017, Zhang *et al.* 2019, Jan *et al.* 2024). Spatial variation in the environment can lead to intraspecific variation in anthocyanin concentration (Vyas *et al.* 2015, Still *et al.* 2023). It can be difficult to discern drivers of this variation, however, since anthocyanins are involved in mitigating the effects of multiple abiotic stressors. In addition to ROS scavenging, anthocyanins can provide photoprotection through light attenuation of blue–green wavelengths that could otherwise lead to ROS production (Hughes and Smith 2007, Fondom *et al.* 2014, Landi *et al.* 2015, Agati *et al.* 2021). Anthocyanin accumulation has been shown in stressful light environments, helping to minimize photoinhibition resulting from absorption of excess photons (Zhu *et al.* 2018, Gould *et al.* 2018).

Aboveground plant architecture, or the spatial arrangement of leaves and branches, is a whole-plant concept highly relevant to light capture (Pearcy and Yang 1996, Valladares and Niinemets 2007, Freschet *et al.* 2018). Architectural adjustments that optimize growth are important for success during early developmental stages (Nascimento *et al.* 2015 , Wei *et al.* 2025). Overlapping leaves contribute to self-shading, which influences light interception and thus has implications for photoprotection and carbon gain (Pearcy *et al.* 2005). Diurnal changes in leaf angle reveal that this trait can be a reversable solution to light management (Nilsen and Forseth 2018). Studies examining the relationship between leaf angle and irradiance have shown that under shade, leaf angle is fairly perpendicular to incoming light for maximized light capture (Planchais and Sinoquet 1998, Ajmi *et al.* 2018). Alternatively, steep leaf angle can be a mechanism to reduce light capture if light intensity exceeds what can be safely processed through the photosynthetic pathway (Pérez-Llorca *et al.* 2019). Despite what is known about leaf angle responses to light, the role of leaf angle in broader ecological processes and its relationships to plant function are not fully recognized (Yang *et al.* 2023).

**Figure 1:**
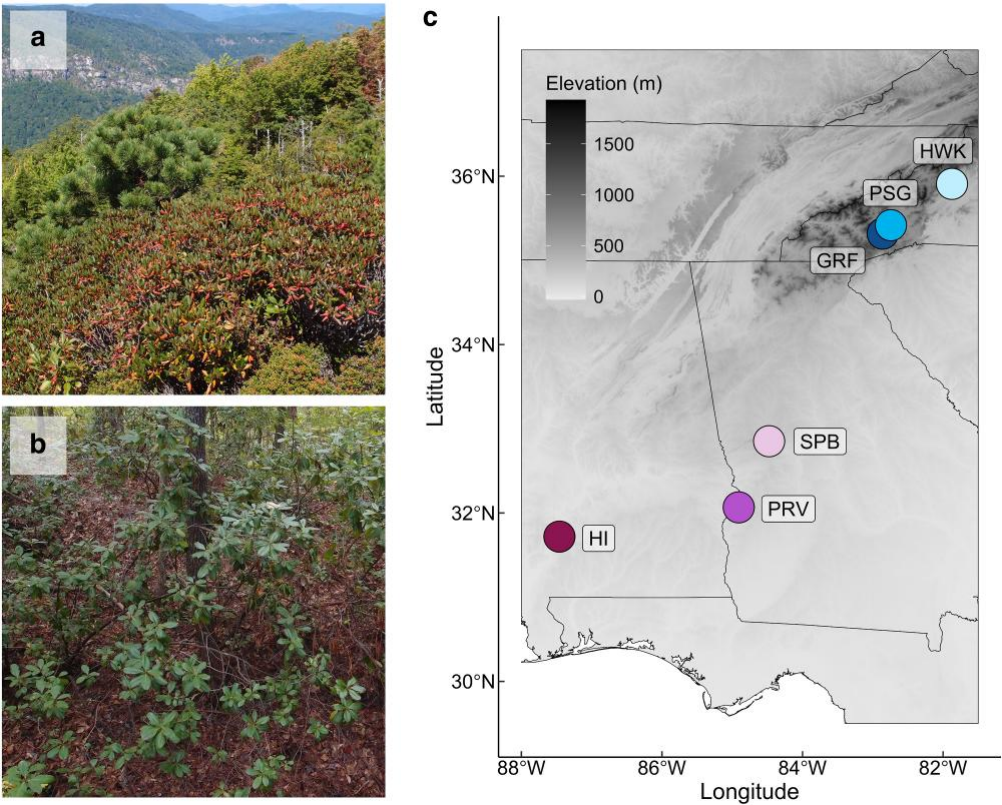
a) *Rhododendron minus* plants growing at Hawkbill Mountain, North Carolina, a sun-exposed location in the Appalachian Mountains. b) *Rhododendron minus* plants growing in the forest understory at Haines Island, Alabama. c) *Rhododendron minus* seed sampling locations across the Southeast United States: Graveyard Fields (GRF), Mount Pisgah (PSG), Hawksbill Mountain (HWK), Sprewell Bluff (SPB), Providence Canyon (PRV), and Haines Island (HI). Seedlings from HWK were used in the manipulative greenhouse experiment, and mature plants from all provenances were used in the common garden experiment. State boundaries and elevation (m) are shown. Photos used by permission from Stephen Krebs.

*Rhododendron minus* is a broadleaf evergreen shrub native to the Southeast United States that grows across a light gradient, from open habitats on rock outcrops (Fig. 1a) to the forest understory (Fig. 1b). This species spans latitudes from eastern Tennessee (∼36° N) to northern Florida (∼30° N; Duncan and Pullen 1962), representing a climate gradient where plants of northern latitudes experience frequent winter frost and snowfall (Konrad and Fuhrmann 2013). *Rhododendron minus* exhibits leaf movements in response to cold temperature (Nilsen 1991), a function that has been suggested to provide winter photoprotection in evergreens (Wang *et al.* 2009, Nilsen *et al.* 2014). Leaf movements are an important morphological adaptation in light management for numerous cold-tolerant *Rhododendron* species; yet, the implications of leaf movements on performance and relevance for survival under contrasting light environments has yet to be fully resolved. Here, we conducted two experiments to elucidate the factors allowing *R. minus* to occupy different abiotic environments, emphasizing the role of light in whole-plant function. First, we conducted a manipulative greenhouse experiment on seedlings grown from seeds collected from a sun-exposed location in the Appalachian Mountains. We expected these seedlings to be highly sensitive to light intensity given field observations of steep leaf angle and the presence of dark green and red leaves on the same plant (Fig. 1a). We examined plant architecture, biomass patterns, and pigments (i.e. chlorophyll and anthocyanin indices) of seedlings under shade, ambient, and supplemental light treatments. Second, we conducted a common garden experiment examining anthocyanin and chlorophyll indices in mature plants from six provenances representing differences in latitude, elevation, solar radiation, and climate (Fig. 1c) with the goal of examining whether environmental factors drive pigment variation.

We asked a series of questions to investigate environmental stress response of *R. minus*. First, do seedlings subjected to supplemental light have more anthocyanin and exhibit larger changes in plant architecture to reduce light interception? We expected these responses to assist with light management under supplemental light. Second, do shaded seedlings have more chlorophyll to increase light capture? This response may help with light absorption for biomass production. Third, is there evidence of intraspecific variation in pigments across the range? This was expected due to climatic and elevational gradients across the species range. Fourth, do plants from provenances characterized by higher light intensity and/or more stressful climates (i.e. colder and drier) exhibit more anthocyanin when grown in a common garden? Given the role of anthocyanins in multiple abiotic stressors (Xu et al. 2017, Zhang et al. 2019, Jan et al. 2024), we hypothesized that the function of anthocyanin in *R. minus* may by shaped by variation in light and climate across the range. In the discussion, we provide context for how leaf movements and anthocyanins may aid in photoprotection of this evergreen species growing at sun-exposed, cold provenances.

## Materials and methods

### Seed collection

In September 2012, *R. minus* seeds were collected and pooled from multiple individuals at six locations, representing different provenances, in the Southeast United States (Fig. 1c): Hawksbill Mountain in the Linville Gorge of North Carolina (HWK: 35.90877° N, 81.87370° W), Mount Pisgah (PSG: 35.41770° N, 82.74212° W) and Graveyard Fields in the Blue Ridge Mountains of North Carolina (GRF: 35.31747° N, 82.85983° W), Sprewell Bluff Wildlife Management Area in Georgia (SPB: 32.85243° N, 84.47512° W), Providence Canyon State Park in Georgia (PRV: 32.06715° N, 84.90723° W), and Haines Island Park in Alabama (HI: 31.71957° N, 87.45873° W). Seeds of the same provenance were stored in a glassine envelope (GLASS-01-1M: Envelopes.com, Melville, NY, USA), which was then placed inside a coin envelope (ESSENTIAL brand). The envelopes, one per provenance, were housed in a single jar with colour-indicating desiccant (W. A. Hammond Drierite Co., Xenia, OH, USA). Desiccant was replaced as needed, and the jar was stored in a freezer until seeds were sown.

### Experiment 1: Manipulative greenhouse experiment

#### Plant material

We studied effects of light intensity on plant architecture, pigments, and biomass patterns using plants grown from seeds collected at Hawksbill Mountain, North Carolina. In October 2023, 625 seeds were sown in a seed starting tray (Bootstrap Farmer, Downingtown, PA, USA) with professional growing mix (Fafard 3B: Sun Gro Horticulture, Agawam, MA, USA) and a layer of peat moss (Premier Horticulture, Quakertown, PA, USA). Seedlings were cultivated in a greenhouse at the Holden Arboretum Long Science Center in Kirtland, Ohio. The greenhouse was equipped with a 50% shade curtain, which was deployed from the time of seed planting to the end of the manipulative experiment. Seedlings experienced natural light patterns in the greenhouse and were misted as needed by hand to maintain well-watered conditions during germination. The greenhouse was temperature-regulated, with a daily mean temperature of 17.70 ± 0.99°C from the time of planting to the start of treatments. In March 2024, 72 seedlings of similar size were haphazardly selected from the emerged seedlings and transplanted into nine cell trays (Grow-Green, Nantong, Jiangsu, China), with each tray containing eight seedlings in separate cells. Seedlings were planted in a 1:1 ratio of sifted pine bark (Gardenscape, Eau Claire, PA, USA) and sifted tree and shrub soil (Sta-Green brand), forming a well-drained, acidic soil for optimal growing conditions of *Rhododendron* species (Krebs 2018). Seedlings were fertilized once every 2 weeks with a foliar application of organic fertilizer (Holly-tone: Espoma, Millville, NJ, USA). Individual seedlings were watered once the top of the soil became dry, creating a wet–dry cycle for the watering regime. Ambient and supplemental light seedlings required the most water and were watered approximately every 2–3 days, respectively, at the minimum.

#### Experimental design

Our greenhouse experiment consisted of three light treatments designed to elicit different responses of *R. minus* seedlings: shade, ambient, and supplemental light. Seedlings in the ambient treatment were exposed to natural light conditions in the greenhouse with the 50% shade curtain deployed. The ambient light environment is what seedlings experienced during seedling cultivation prior to subjection to shade or supplemental light. To establish the shade treatment, we placed seedlings under a wooden frame fitted with a single layer of 50% shade cloth (AgFabric, Wellco Industries Inc., Corona, CA, USA), further reducing irradiance beyond that of the ambient light environment. The supplemental light treatment was established by hanging a 1000 W full spectrum grow light (Shenzhen Hanhua Optoelectronics Co., Ltd., Shenzhen, Guangdong, China) over the seedlings, providing high quantities of red (620–630 nm) and blue light (450–460 nm). Treatments were replicated in a stratified design consisting of three blocks, with eight seedlings per treatment–block combination: 8 replicates × 3 treatments × 3 blocks = 72 seedlings. Treatments began on 1 April 2024 and lasted 12 weeks until seedlings were harvested on 25 June 2024. Temperature and relative humidity were recorded at one location in the greenhouse every 300 s by a sensor (S-THB-M002: Onset, Bourne, MA, USA) connected to a HOBO datalogger (U30: Onset, Bourne, MA, USA). Mean temperature and relative humidity throughout the experiment were 21.39 ± 3.05°C and 62.34 ± 11.91%, respectively.

#### Light intensity measurements

To characterize light availability of each treatment, photosynthetic photon flux density (PPFD) was logged every 300 s throughout the experiment using three HOBO datalogger photosynthetically active radiation (PAR) sensors (S-LIA-M003: Onset, Bourne, MA, USA), with one sensor per treatment. The sensors were positioned directly adjacent to seedlings of each treatment in the first block. We calculated daily light integral (DLI) of each treatment as the sum of PPFD across each 24-hour day multiplied by the 300 s time interval. We also determined daily maximum PPFD recorded by each PAR sensor to characterize light peaks in the greenhouse.

To assess variation in irradiance among plant replicates, we measured PPFD on an overcast day at solar noon, immediately after the conclusion of the experiment, using a hand-held light meter (LI-250A: LICOR Inc., Lincoln, NE, USA) with a quantum sensor attached (Li-190R: LICOR Inc., Lincoln, NE, USA). We conducted spot measurements, recording one 15-s mean PPFD at each plant location at tray height.

#### Change in projected area

To understand how treatment influenced the amount of surface area available for direct light interception, we compared aboveground projected area at two timepoints as a method to quantify leaf movements. On 29 May 2024, we photographed each seedling from an aerial perspective ∼8 cm above the plant against cardstock to assist with background subtraction during image processing. On 24 June 2025, 1 day before harvest, seedlings were photographed from a similar orientation as the initial image. Projected area (cm^2^) was calculated in ImageJ for each plant at both timepoints as projected area_initial_ and projected area_final_. An absolute change in projected area was calculated as the difference between the final and initial measures. To account for potential influences of plant size on change in projected area, we divided the absolute change by total leaf area at the time of harvest (see biomass trait measurement below) as follows: (projected area_final_ − projected area_initial_)/total leaf area. We did not expect substantial growth from the end of May to the time of harvest at the end of June since *R. minus* is a slow-growing, evergreen plant that exhibits a flush of new growth in the spring. Furthermore, leaf out has been shown to occur during April and May for a different *Rhododendron* species from the Appalachian Mountains (Lei *et al.* 2006), and so we expected our initial and final time points to be after the newest flush of leaves. Since *R. minus* is known to exhibit leaf movements in response to the environment, changes in projected area were expected to primarily reflect changes in plant architecture, where a positive change suggests an increase in projected surface area over time and a negative change suggests a decrease.

#### Multispectral imaging

Based on Wacker *et al.* (2024), we constructed a low-cost multispectral imaging device to calculate two reflectance indices related to plant pigments, the normalized difference anthocyanin index (NDAI), which uses reflectance under green and red light to estimate anthocyanins (Kim and van Iersel 2023), and the normalized difference vegetation index (NDVI), which uses reflectance under red and near-infrared light to estimate chlorophyll (Li *et al.* 2018, Wacker *et al.* 2024). Our device was constructed from a Raspberry Pi 4B computer (Raspberry Pi Foundation, Cambridge, England, United Kingdom) that acquires images from a USB monochrome camera module (B0332: Arducam, Nanjing, Jiangsu, China) under three types of light-emitting diodes (LEDs): green (iNextStation brand), red (iNextStation brand), and near-infrared (360DigitalSignage brand). We adhered two 25 cm long LED strips of each type above a 300 × 300 × 2 mm light-diffusing acrylic sheet (Sevenneonlighting, Shenzhen, Guangdong, China), ensuring uniform light distribution during imaging (Wacker *et al.* 2024 ). The camera module was positioned through a 15 mm hole in the centre of the acrylic sheet, which was fastened above a wire rack where plants were placed on for imaging. The rack was positioned inside of a lightproof grow tent (GreenHouser brand). We wired the LED strips to a breadboard with a 12 V power supply and used metal oxide semiconductor field-effect transistors (MOSFETs: ALLECIN brand) to act as a switch for controlling voltage to the LEDs.

We wrote a multispectral imaging script in Python version 3.11.2 that automated the acquisition of images under each type of LED with periodic checkpoints to ensure proper LED function. After photographing the plant under each type of LED, the script then prompted the user to open the grow tent so that an image of the plant could be taken under natural light for background subtraction. We wrote an ImageJ macro (version 1.54 g) that created a mask of each plant from the natural light image and used the mask to extract mean pixel intensity (*i*) from the green, red, and near-infrared images. We calculated NDAI as (*i*_red_ – *i*_green_)/(*i*_red_ + *i*_green_) and NDVI as (*i*_near-infrared_ – *i*_red_)/(*i*_near-infrared_ + *i*_red_). We used mean pixel intensities rather than calculating reflectance indices at each pixel because our experiment was designed to investigate variation in pigments across plants rather than within plants. We conducted multispectral imaging on each seedling at the end of the experiment on 24 June 2024.

#### Biomass

Seedling survival was recorded at the end of the experiment on 25 June 2024 and plants were subsequently harvested. Leaves were removed from each seedling and photographed for calculation of total leaf area in ImageJ. Roots were preserved in SafeFix (Thermo Fisher Scientific, Waltham, MA, USA) for up to 4 weeks until they were processed using WinRHIZO version Pro 2013e (Regent Instruments, Quebec City, Quebec, Canada) to obtain root length. Leaf, stem, and root biomass were dried for a minimum of 72 hours at 60°C and subsequently weighed. Specific leaf area (SLA: cm^2 ^mg^−1^) was calculated as the ratio of one-sided leaf area to dry leaf biomass; specific root length (SRL: cm mg^−1^) was calculated as the ratio of root length to dry root biomass; root to shoot ratio (R:S) was calculated as the ratio of dry belowground to dry aboveground biomass; and total biomass (mg) was calculated as the sum of dry leaf, stem, and root biomass. A linear regression with R:S as the response variable and total biomass and treatment as predictor variables showed no significant main effect of total biomass nor a significant interaction between treatment and total biomass, indicating that development did not differentially influence biomass partitioning between belowground and aboveground tissues (McConnaughay and Coleman 1999).

### Experiment 2: Common garden experiment

#### Plant material

To complement our manipulative greenhouse experiment, which included seedlings from only one provenance, we compared plants from the six seed collection locations described above. Seeds were germinated in 2013, after which plants were transferred in individual pots to grow at Holden Arboretum Long Science Center starting in 2016. Cuttings of mature plants were collected in October 2021 and grown in a series of pots ranging from 30.5 to 50.8 cm using a mix of Fafard 3B (Sun Gro Horticulture, Agawam, MA, USA) and composted hardwood mulch. Plants were fertilized with Holly-tone (Espoma, Millville, NJ, USA) in the spring and fall of each year. From April to November, plants were grown outside in a lath house, meaning a structure with evenly positioned wooden slats that provide 50% shade of natural light and exposure to natural weather patterns. Plants were supplemented with additional water by hand if rainfall was not sufficient to maintain a wet–dry cycle. From December to March, plants were placed in a polyhouse where they were held above 12°C and received natural light filtered through a white plastic tarp. Plants from six provenances were organized in a stratified random design and frequently rotated to account for environmental heterogeneity in the common garden.

#### Experimental design

In July 2024, there was one genotype per provenance available for sampling, and each genotype had multiple rooted cuttings available for sampling. We sampled six cuttings per genotype for all provenances except for Mount Pisgah for which we only had four cuttings. We haphazardly chose one uppermost fully expanded leaf from each cutting for pigment estimation. Leaves were photographed using the same multispectral imaging device from our manipulative greenhouse experiment, and NDAI and NDVI were calculated as described above.

#### Provenance abiotic environment

To understand relationships of pigment indices to environmental variables, we obtained elevation data derived from the Shuttle Radar Topography Mission (SRTM), solar radiation data, and 19 bioclimatic variables related to temperature and precipitation from the WorldClim version 2 database at a 30 arc-second spatial resolution (Fick and Hijmans 2017). We excluded mean temperature of the wettest quarter and driest quarter because these variables have been shown to have large spatial discontinuities in the Southeast United States that are likely artefacts of the way they were generated (Booth 2022). We calculated mean annual solar radiation by averaging across monthly data. Environmental data were extracted from the geographic coordinates of each provenance using the *terra* R package (Hijmans 2025).

### Statistical analyses

#### Experiment 1: Manipulative greenhouse experiment

To examine whether our treatments were successful in delivering different light intensities, we used the ‘lmer’ function from the *lme4* R package (Bates *et al.* 2015), within R version 4.4.1, to construct mixed-effects models with light treatment as the predictor variable, block as a random effect, and PPFD measured on an overcast day as the response variable. To examine trait differences across light treatments, we constructed similar mixed-effects models with NDAI, NDVI, change in projected area, SLA, SRL, R:S, or total biomass as the response variable. Some mixed-effects models had a singular fit, indicating that variance explained by block was zero or nearly zero. In these cases, the models were reduced to simple linear regressions without block as a random effect. We used analysis of variance (ANOVA) to test for a significant treatment effect in linear mixed-effects models and simple linear models, implementing the ‘Anova’ function from the *car* R package (Fox and Weisberg 2019). When ANOVA indicated a significant treatment effect, we used the *emmeans* R package (Lenth 2025 ) to examine whether there were significant pairwise differences across treatments. The Tukey method was used to account for multiple comparisons. We used the ‘glm’ function from the *stats* R package (R Core Team 2024) to examine the influence of light treatment on survival, constructing a generalized linear model with treatment as the predictor variable and survival as the binary response variable. The model with block had a singular fit, so we report the reduced model.

#### Experiment 2: Common garden experiment

We tested for intraspecific variation in NDAI and NDVI by conducting an ANOVA across all measured leaves with the ‘aov’ function from the *stats* R package (R Core Team 2024). For these tests, provenance was the predictor variable and a pigment index was the response variable. When ANOVA indicated a significant effect of provenance, we used the ‘TukeyHSD’ (Tukey Honest Significant Difference) function from the *stats* R package (R Core Team 2024) to compute pairwise differences across provenances. Since only one genotype per provenance was available for sampling, we also calculated mean pigment indices for each provenance and sorted provenances into two groups based on geographic location. Graveyard Fields, Mount Pisgah, and Hawksbill Mountain reside in the northern region of the species range and have higher elevations in the Appalachian Mountains than southern provenances, namely Sprewell Bluff, Providence Canyon, and Haines Island (Fig. 1c). Furthermore, these northern provenances belong to USDA hardiness zone 7, whereas southern provenances belong to zone 8 (USDA Plant Hardiness Zone Map 2023). With this natural geographic and climatic separation of provenances, we included Graveyard Fields, Mount Pisgah, and Hawksbill Mountain as ‘northern’ provenances and Sprewell Bluff, Providence Canyon, and Haines Island as ‘southern’ provenances. Using the ‘aov’ function, we set ‘northern’ versus ‘southern’ as the predictor variable and a mean pigment index as the response variable. We also examined relationships between environmental variables and pigment means across provenances. To characterize provenance climate, we conducted a principal component analysis (PCA) using the ‘prcomp’ function from the *stats* R package (R Core Team 2024) with arguments ‘scale’ and ‘center’ = TRUE, reducing the dimensionality of the 17 climate variables to the first and second principal components (PC1 and PC2). A Pearson correlation matrix was calculated using the ‘cor’ function from the *stats* R package (R Core Team 2024) and included elevation, solar radiation, MAT, temperature seasonality, annual precipitation, precipitation seasonality, PC1, PC2, mean NDAI, and mean NDVI. We do not report correlations among climate variables since these relationships are shown in the PCA. We examined significance of the correlations and visualized the matrix using the ‘cor.mtest’ and ‘corrplot’ functions, respectively, from the *corrplot* R package (Wei and Simko 2024 ).

## Results

### Experiment 1: Manipulative greenhouse experiment

#### Light intensity measurements

For PAR sensors placed in shade, ambient, and supplement light, daily light integral (DLI) means and standard deviations were 2.27 ± 0.93 mol m^−2^ d^−1^, 4.62 ± 1.71 mol m^−2^ d^−1^, and 17.24 ± 2.81 mol m^−2^ d^−1^, respectively, whereas daily maximum PPFD means and standard deviations were 486 ± 135 µmol m^−2^ s^−1^, 1514 ± 466 µmol m^−2^ s^−1^, and 1456 ± 273 µmol m^−2^ s^−1^, respectively. A mixed-effects model of PPFD measured across all plant locations on an overcast day revealed a significant treatment effect on PPFD (χ^2^ = 10 411.43, *P* < 0.001), and a *post hoc* Tukey test showed significant differences across all pairwise comparisons (*P* < 0.001). Mean (±standard error) PPFD for shade, ambient, and supplement light treatments were 8.53 ± 0.77 μmol m^−2^ s^−1^, 28.46 ± 1.85 μmol m^−2^ s^−1^, and 207.10 ± 1.95 μmol m^−2^ s^−1^, respectively.

#### Influence of light treatment on traits

**Figure 2:**
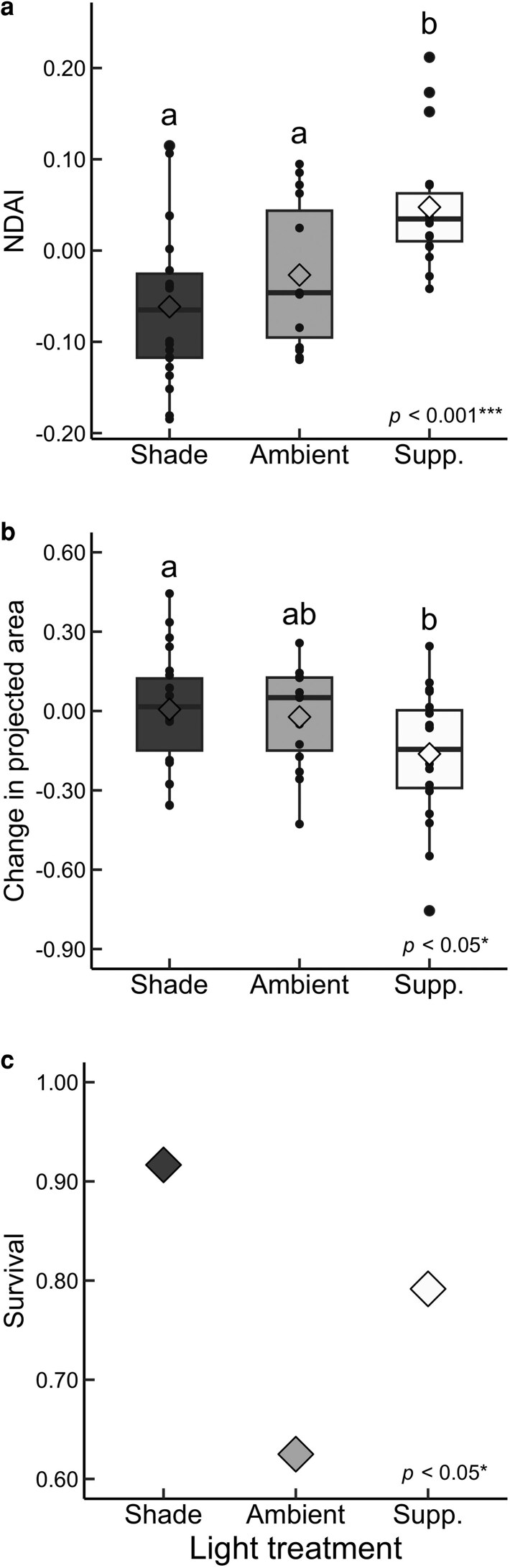
Plots of *Rhododendron minus* seedlings from Hawksbill Mountain, North Carolina across three light treatments, shade, ambient, and supplemental light: a) normalized difference anthocyanin index (NDAI); b) change in projected area (ratio of absolute change to total leaf area); c) survival percentage out of 24 seedlings per treatment group. Each box plot consists of the median as the thick horizontal line along with the lower and upper hinges representing the first and third quartiles, respectively. The whiskers extend from the hinges to data within 1.5× interquartile range. The mean for each treatment is indicated by a diamond. All data points are shown overlaying the box plots. Letters represent significant pairwise differences according to the Tukey method for multiple comparisons. Sample sizes for shade, ambient, and supplemental light treatments for NDAI and change in projected area were 22, 15, and 19, respectively, which reflect differences in survival during the experiment.

We found a significant treatment effect on NDAI, such that plants in the supplement light treatment had significantly higher mean NDAI than plants in ambient and shade conditions (Fig. 2a, Table 1). There was also a significant treatment effect on change in projected area, with a near-zero mean for shade seedlings, a slight mean decrease for ambient seedlings, and a larger mean decrease for supplemental light seedlings (Fig. 2b, Table 1). A *post hoc* Tukey test revealed that supplemental light seedlings had significantly larger change in projected area than shade seedlings, with the change being in the negative direction. A generalized linear model revealed a significant treatment effect on survival (Table 1). Shade seedlings had the highest survival, supplemental light seedlings had intermediate survival, and ambient seedlings had the lowest survival, but no significant pairwise differences were detected (Fig. 2c). We did not find significant treatment effects on NDVI, SLA, SRL, R:S, or total biomass (Tables 1 and  2).

**Table 1:** Summary of ANOVA models testing for significance of light treatment, shade, ambient, and supplemental light, on traits of *Rhododendron minus* seedlings from Hawksbill Mountain, North Carolina.

Trait	Model type	Test statistic	*P*
NDAI	Mixed effects	25.80	<0.001***
NDVI	Simple linear	0.23	0.797
Change in projected area	Mixed effects	6.92	**0**.031*
SLA	Mixed effects	5.73	0.057
SRL	Mixed effects	0.86	0.650
Total biomass	Mixed effects	0.60	0.742
R:S	Simple linear	0.04	0.956
Survival	Generalized linear	6.19	**0**.045*

The test statistic for mixed-effects models and the generalized linear model is χ^2^, while *F* is the test statistic for the simple linear models. Bold indicates significance: *P < 0.05, ***P < 0.0001.

**Table 2:** Means and standard errors of traits related to chlorophyll and biomass for *Rhododendron minus* seedlings from Hawksbill Mountain, North Carolina across three light treatments, shade, ambient, and supplemental light: normalized difference vegetation index (NDVI), specific leaf area (SLA, cm^2^ mg^−1^) specific root length (SRL: cm mg^−1^), total biomass (mg), and root to shoot ratio (R:S).

Trait	Shade	Ambient	Supplemental
NDVI	0.18 ± 0.01	0.17 ± 0.02	0.17 ± 0.02
SLA	0.25 ± 0.01	0.24 ± 0.02	0.20 ± 0.02
SRL	30.51 ± 3.60	36.99 ± 6.80	31.07 ± 6.16
Total biomass	3.89 ± 0.68	4.78 ± 1.44	4.75 ± 0.84
R:S	0.22 ± 0.04	0.22 ± 0.04	0.24 ± 0.04

Sample sizes for shade, ambient, and supplemental light treatments were as follows, respectively: NDVI and SLA (22, 15, and 19), SRL (20, 15, 17), total biomass (22, 15, 18), and R:S (22, 15, 18).

### Experiment 2: Common garden experiment

#### Influence of provenance on pigments

**Figure 3:**
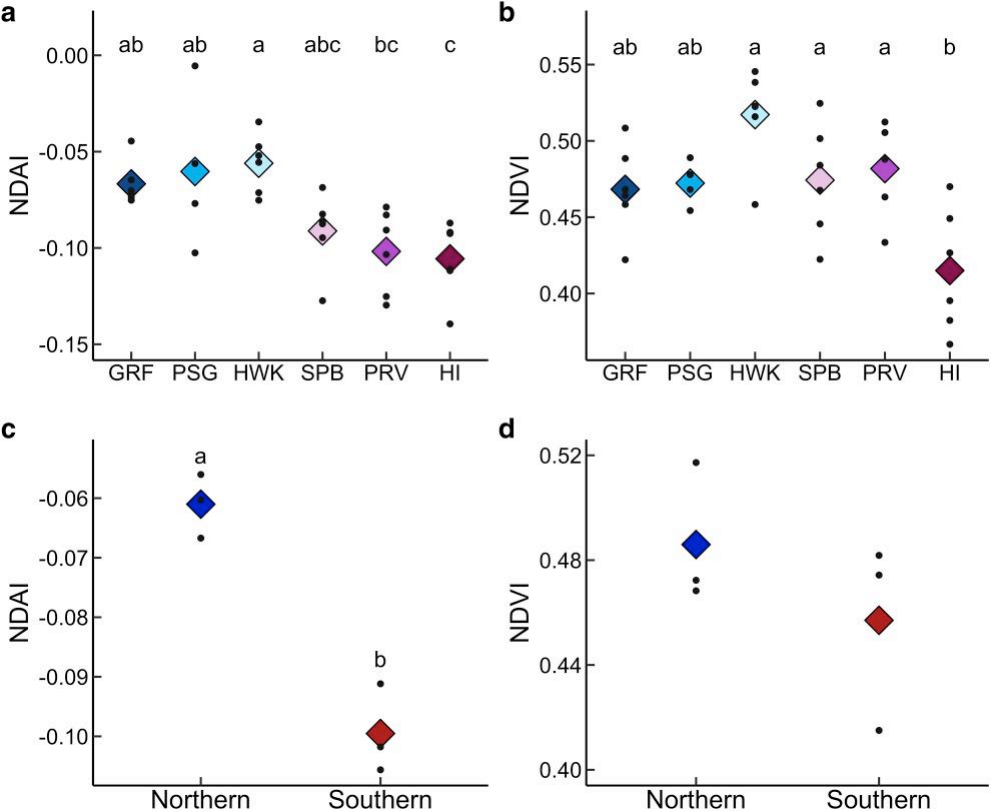
Normalized difference anthocyanin index (NDAI) and normalized difference vegetation index (NDVI) across six *Rhododendron minus* provenances (a and b), and across northern and southern provenances (c and d). Means are shown as diamonds, and all data points are shown. a, b) Letters represent significant pairwise differences according to the Tukey method for multiple comparisons. Sample size was six cuttings for all provenances except for Mount Pisgah which had a sample size of four cuttings. c, d) Letters represent a significant difference between northern and southern provenances. Northern provenances include Graveyard Fields (GRF), Mount Pisgah (PSG), and Hawksbill Mountain (HWK), and southern provenances include Sprewell Bluff (SPB), Providence Canyon (PRV), and Haines Island (HI), resulting in a sample size of three for both northern and southern provenances.

There was a significant effect of provenance on NDAI (*F* = 5.79, *P* < 0.001) with a *post hoc* Tukey test showing that mean NDAI of Graveyard Fields, Mount Pisgah, and Hawksbill Mountain were significantly higher compared to Haines Island (Fig. 3a). Mean NDAI of Hawksbill Mountain was also significantly higher than Providence Canyon (Fig. 3a). Similarly, ANOVA showed a significant effect of provenance on NDVI (*F* = 6.27, *P* < 0.001). Haines Island had the lowest mean NDVI, which was significantly lower than mean NDVI of Hawksbill Mountain, Sprewell Bluff, and Providence Canyon (Fig. 3b). We also found significantly higher NDAI for northern provenances compared to southern provenances (*F* = 52.40, *P* = 0.002; Fig. 3c) but no significant difference in NDVI (*F* = 1.21, *P* = 0.33; Fig. 3d).

#### Provenance environment

**Figure 4:**
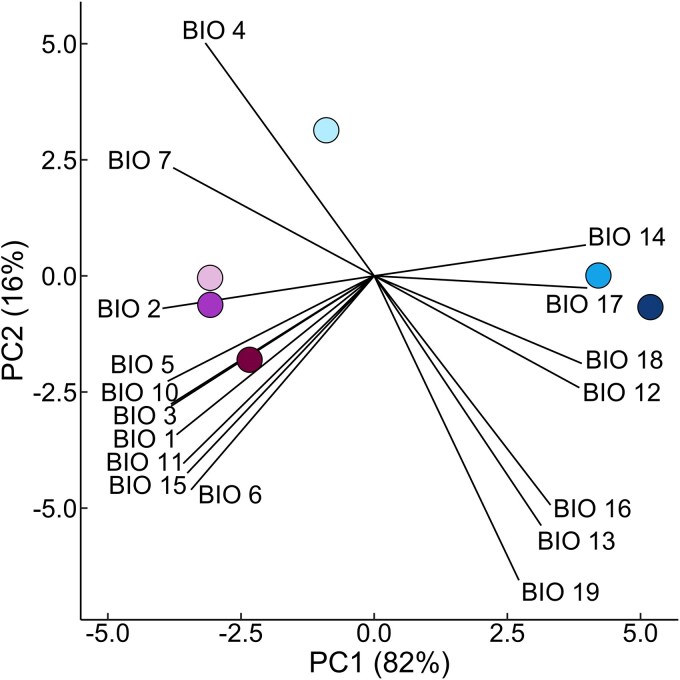
Principal component analysis (PCA) of 17 bioclimatic variables across the six *Rhododendron minus* provenances used in the common garden experiment. Variables include the following: BIO1 = mean annual temperature, BIO2 = mean diurnal range [mean of monthly (max. temp.–min. temp.)], BIO3 = isothermality [(BIO2/BIO7) × 100], BIO4 = temperature seasonality (standard deviation × 100), BIO5 = maximum temperature of the warmest month, BIO6 = minimum temperature of the coldest month, BIO7 = temperature annual range (BIO5–BIO6), BIO10 = mean temperature of the warmest quarter, BIO11 = mean temperature of the coldest quarter, BIO12 = annual precipitation, BIO13 = precipitation of the wettest month, BIO14 = precipitation of the driest month, BIO15 = precipitation seasonality (coefficient of variation), BIO16 = precipitation of the wettest quarter, BIO17 = precipitation of the driest quarter, BIO18 = precipitation of the warmest quarter, BIO19 = precipitation of the coldest quarter. Points represent the provenances, with the colour of each provenance shown in Fig. 1c. Note that PC loadings were scaled by a factor of 15 for visualization purposes.

The first and second principal components explained 82% and 16% of variation in climate among provenances, respectively (Fig. 4). The first principal component (PC1) was driven strongly in the positive direction by precipitation of the driest quarter and driest month and strongly in the negative direction by mean diurnal range. Smaller positive contributions to PC1 included precipitation of the warmest quarter and annual precipitation, with smaller negative contributions of maximum temperature of the warmest month, mean temperature of the warmest quarter, isothermality, temperature annual range, and MAT. The three southern provenances were distinguished from Mount Pisgah and Graveyard Fields along PC1. The second principal component (PC2) was positively driven by temperature seasonality and negativity driven by precipitation of the coldest quarter, wettest month, and wettest quarter. Precipitation seasonality, mean temperature of the coldest month, and minimum temperature of the coldest quarter had smaller negative contributions to PC2. Hawksbill Mountain was separated from all other provenances along PC2, characterized by higher temperature seasonality and lower precipitation of the coldest and wettest quarters (Fig. 4).

**Figure 5:**
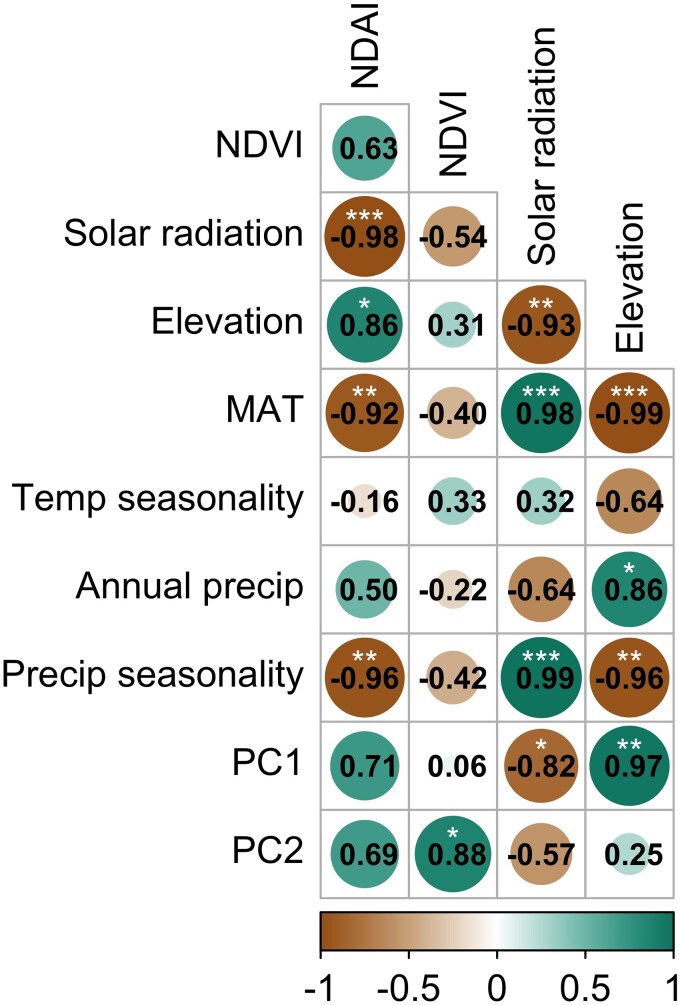
Pearson correlation analysis of pigment indices and environmental variables from six *Rhododendron minus* provenances used in the common garden experiment. Teal and brown shades indicate positive and negative correlations, respectively. Significance levels are shown: *P < 0.05, **P < 0.01, ***P < 0.0001. Variables include normalized difference anthocyanin index (NDAI), normalized difference vegetation index (NDVI), mean annual solar radiation (solar radiation, kJ m^−2^ day^−1^), elevation (m), mean annual temperature (MAT, °C), temperature seasonality (temp. seasonality, standard deviation × 100), annual precipitation (annual precip., mm), precipitation seasonality (precip. seasonality, coefficient of variation), and the first and second principal components (PC1 and PC2, respectively).

We found significant correlations between NDAI and multiple environmental variables (Fig. 5). There was a significant positive correlation between NDAI and elevation and significant negative correlations between NDAI and solar radiation, MAT, and precipitation seasonality. We detected high correlation among these four environmental variables (Fig. 5). Provenances with lower MAT (i.e. ‘northern’ provenances) had less solar radiation, higher elevations, and lower precipitation seasonality (Table 3). A positive correlation between NDVI and PC2 was the only significant correlation for this pigment index (Fig. 5). Furthermore, we did not find a significant correlation between NDAI and NDVI.

**Table 3:** Geographic coordinates in decimal degrees and environmental variables extracted from six *Rhododendron minus* provenances.

	Latitude	Longitude	Solar radiation	Elevation	MAT	Temp. season.	Annual precip.	Precip. season.
GRF	35.32	−82.86	14772	1607	8.94	644.17	1968	8.48
PSG	35.42	−82.74	14 814	1515	9.43	656.84	1855	8.47
HWK	35.91	−81.87	14 813	885	11.72	724.27	1322	10.48
SPB	32.85	−84.48	15 901	206	17.05	705.63	1284	20.31
PRV	32.07	−84.91	16 103	190	17.54	684.34	1283	23.12
HI	31.72	−87.46	16 205	73	18.08	690.91	1476	21.97

Environmental variables include mean annual solar radiation (solar radiation, kJ m^−2^ day^−1^), elevation (m), mean annual temperature (MAT, °C), temperature seasonality (temp. season., standard deviation × 100), annual precipitation (annual precip., mm), and precipitation seasonality (precip. season., coefficient of variation). Northern provenances include Graveyard Fields (GRF), Mount Pisgah (PSG), and Hawksbill Mountain (HWK), and southern provenances include Sprewell Bluff (SPB), Providence Canyon (PRV), and Haines Island (HI).

## Discussion

We examined traits underlying success of *R. minus* across light environments, helping to elucidate strategies for environmental tolerance. This work builds upon previous understanding of plant light responses by showing combined relevance of leaf movements and anthocyanin in light management, helping seedlings maintain similar chlorophyll and total biomass across treatments. Although our greenhouse experiment showed higher NDAI for supplemental light seedings, our common garden experiment showed a negative correlation between NDAI and solar radiation across provenances. It is likely that increased NDAI in northern provenances is advantageous in exposed, cold habitats of the Appalachian Mountains.

Our manipulative greenhouse experiment revealed that supplemental light seedlings had significantly higher NDAI than shade and ambient seedlings (Fig. 2a), which was expected due to the role of anthocyanins in photoprotection (Zhu *et al.* 2018, Zheng *et al.* 2021). Interestingly, there was no significant difference in NDAI between shade and ambient seedlings despite significantly higher PPFD for ambient seedlings. This is likely due to greater similarity of the shade and ambient light environments compared to the supplemental light environment. Supplemental light seedlings had 7.3-fold and 24.3-fold larger mean PPFD compared to ambient and shade seedlings, respectively, whereas ambient seedlings experienced only 3.3-fold larger mean PPFD than shade seedlings. This strong influence of light on anthocyanin accumulation provides robust evidence for the importance of anthocyanins in light management (Gould *et al.* 2018, Davies *et al.* 2022).

To accompany our greenhouse experiment on seedlings from one provenance, we also examined pigment differences across the species range. Our common garden experiment revealed evidence of intraspecific variation in NDAI of mature plants reflecting differences in environmental factors. Northern provenances had higher NDAI compared to southern provenances (Fig. 3c), with NDAI being positively correlated with elevation and negatively correlated with solar radiation, precipitation seasonality, and MAT (Fig. 5). These four environmental variables were highly correlated, which is not surprising since latitude, climate, and solar radiation are known to be highly interdependent (Budyko 1969, Hawkins and Diniz-Filho 2004, Trenberth and Shea 2005, Teke *et al.* 2015). This does, however, make it difficult determine drivers of anthocyanin variation. Given the role of anthocyanins in multiple abiotic stressors (Yan *et al.* 2022) and variation in potential selective factors across the range, *R. minus* presents an interesting system for future investigation of pigment evolution.

Light stress associated with high elevations has been related to increased anthocyanin (Zhao *et al.* 2023). Although elevation was positively correlated with NDAI, solar radiation was negatively correlated with NDAI (Fig. 5), likely due to greater cloud cover and higher latitudes of northern provenances (Körner 2003, Fick and Hijmans 2017). This seems to contradict results of our greenhouse experiment as well as other studies showing a positive association between light intensity and anthocyanins (Lu *et al.* 2015, Stetsenko *et al.* 2020, An *et al.* 2023). It is important to note, however, that the measure of solar radiation used in this study does not account for heterogeneity in light due to the forest canopy; yet, *R. minus* plants in the south may be more likely to experience variable light conditions in the understory compared with plants in the north that tend to grow in open habitats on rock outcrops (Fig. 1a and b; Wharton 1978, Newell and Peet 1998). Canopy gaps may cause periods of intense light leading to temporary overexcitation of the photosynthetic apparatus (Feng *et al.* 2002, Smith and Berry 2013), and so anthocyanins have been suggested to confer photoprotection in understory plants (Hughes *et al.* 2008, Zhu *et al.* 2016, Poobathy *et al.* 2018). Interestingly, in our greenhouse experiment, supplemental light seedlings had significantly higher NDAI than ambient seedlings despite comparable daily maximum PPFD, indicating that sunflecks may be less relevant to anthocyanin production in *R. minus*. Future investigation of the role of pigments in the understory should involve PAR sensor deployment to characterize fine-scale light heterogeneity. The negative correlation between NDAI and MAT suggests that anthocyanin may be beneficial in colder environments (Fig. 5). Plants from northern provenances may benefit from higher NDAI since light stress can be exacerbated in overwintering leaves as photosynthesis is downregulated due to low temperature (Gould *et al.* 2018, Liu *et al.* 2020, Chang *et al.* 2021, Yu *et al.* 2021). Similarly, intraspecific variation in anthocyanin has been attributed to temperature differences across a latitudinal gradient (Vyas *et al.* 2015, Still *et al.* 2023). Taken together, the two experiments indicate that anthocyanin variation within *R. minus* may be driven by combined pressures of exposed, montane habitats and low temperature in the north, whereas southern provenances may rely more heavily on carotenoids, a separate photoprotective pigment system (Klem *et al.* 2015, Sun *et al.* 2022, Simkin *et al.* 2022).

Unlike NDAI, we did not see a significant difference in NDVI between northern and southern provenances, which may indicate different drivers of variation in anthocyanin and chlorophyll for this species. We found no relationship between NDVI and solar radiation across provenances (Fig. 5), supporting findings from our manipulative greenhouse experiment showing NDVI to be independent of light treatment (Table 1). Under high light intensity, chlorophyll content has been shown to decrease with increased anthocyanin synthesis (Singh *et al.* 2017, Qin *et al.* 2024). Interestingly, Zheng *et al.* (2021) found that plants with light-induced anthocyanin production did not decrease chlorophyll content as severely as plants with less anthocyanin content, suggesting that anthocyanins function as light attenuators to shield chlorophyll from potentially damaging light intensities. Although additional research is needed to determine the relative contribution of ROS scavenging and light attenuation to the functional significance of anthocyanins in *R. minus*, our results suggest that chlorophyll is maintained under different light intensities that are suitable for growing. This may improve energy balance, as biosynthesis of anthocyanin has been associated with an energy cost and hence attributed to a growth trade-off (Yu *et al.* 2019).

In addition to influence of light treatment on NDAI, we found a significant treatment effect on change in projected area that we attribute to architectural adjustments. Specifically, we observed pendent, overlapping leaves in supplemental light seedlings, contributing to a decrease in projected area over time (Fig. 2b). These leaf movements should reduce direct light capture (Falster and Westoby 2003, Nagler *et al.* 2004), helping to balance light interception with metabolic capacity. Thermonasty, meaning leaf movements in response to temperature, has been suggested to aid in photoprotection of temperate broadleaf evergreen *Rhododendron* species given joint stressors of cold temperature and high irradiance limiting photosynthetic capacity during winter months (Nilsen *et al.* 2014). Moreover, *Rhododendron* leaves have been observed to be more pendant under deciduous canopies that allow greater irradiance to reach the understory (Nilsen 1985). Our study builds upon this by showing experimental evidence of whole-plant architectural adjustments under contrasting light treatments, helping to isolate light intensity as a variable driving plant stress response.

Greater light availability can lead to higher photosynthetic rate (Feng *et al.* 2019) and thus greater biomass production (Chen *et al.* 2023) and faster growth rate (Rugemalila *et al.* 2020), but whether these benefits are realized depends on whether plants balance light absorption with metabolic capacity (Wimalasekera 2019). As a slow-growing, shade-tolerant evergreen species with relatively low metabolic capacity and conservative resource-use (Salgado-Luarte and Gianoli 2017, Medeiros *et al.* 2022), our results suggest that *R. minus* seedlings accumulate anthocyanin and adjust leaf angle to manage light stress. Seedlings from Hawksbill Mountain may be adequately suited to a range of light environments given similar performance across the light intensities examined here. Complex interactions between irradiance and climate may be driving intraspecific variation in pigments across provenances, emphasizing the importance of multiple environmental factors in plant response.

## Data Availability

Raw data and R code are available online at https://github.com/mkshetzer/2026_light_ms.git. Also included are a Python script for the multispectral imaging device and an ImageJ macro for multispectral image analysis of the manipulative greenhouse experiment.
